# INTERDISCIPLINARY CURRICULUM FOR THE CARE OF OLDER ADULTS: CREATING NETWORKS OF COLLABORATIVE LEARNERS

**DOI:** 10.1093/geroni/igz038.1082

**Published:** 2019-11-08

**Authors:** Pamela Yankeelov, Anna Faul, Joe D'Ambrosio, and Samantha G Cotton

**Affiliations:** University of Louisville, Louisville, Kentucky, United States

## Abstract

Serving older adults with multiple chronic conditions and variable social, emotional, or physical support effectively within the primary care setting requires an interdisciplinary approach to care. Our GWEP program has developed an interprofessional education center that educates and prepares students and professionals from social work, medicine, nursing, dentistry, pharmacy, and community health partners, to function within a transformed integrated patient-centered geriatric primary care and community-based service delivery system. Learners from multiple disciplines attend a face-to-face Interdisciplinary Case Management Experience (ICME) session lasting 2.5 hours. Sessions include learners from each discipline and, if possible, at least one community practitioner in small groups of 6–8 learners at each table facilitated by 1 faculty member. Approximately 1,200 learners have received the curriculum. To evaluate the program, Kirkpatrick’s Training Evaluation Model was used to determine if learners were satisfied with the content, skilled, and confident in their abilities to utilize the curriculum. Learners completed a satisfaction survey after taking each module, along with an interdisciplinary geriatric care knowledge test and self-efficacy test before and after taking each module to measure learning outcomes. Analysis showed that learners, irrespective of discipline, were satisfied with the program. All disciplines showed a significant increase from pre- to posttest for all 5 online modules achieving a mean post-knowledge score of 85% across all 5 online training modules. All disciplines experienced significant differences in their self-efficacy with working on interdisciplinary teams from pre to post ICME. Implications for future interprofessional curriculum will be discussed.

